# Sight threatening diabetic retinopathy in patients with macular telangiectasia type 2

**DOI:** 10.1186/s40942-024-00545-5

**Published:** 2024-03-12

**Authors:** Josef Huemer, Tjebo FC Heeren, Abraham Olvera-Barrios, Livia Faes, Antonio M. B. Casella, Edward Hughes, Adnan Tufail, Catherine Egan

**Affiliations:** 1 Moorfields Eye Hospital, NHS Foundation Trust, 62 City Rd., EC1V 2PD London, UK; 2 Department of Ophthalmology and Optometry, Kepler University Hospital, Linz, Austria; 3 University College London Institute of Ophthalmology, London, UK; 4 Department of Surgery, Health Sciences Center, Londrina State University, Paraná, Brazil; 5 University Hospitals Sussex NHS Foundation Trust, Brighton, UK

**Keywords:** Macular telangiectasia type 2, Proliferative diabetic retinopathy, Sight threatening diabetic retinopathy, Epiretinal neovascularization, OCT angiography, Fluorescein angiography

## Abstract

**Purpose:**

Although diabetes is highly prevalent in patients with macular telangiectasia type 2 (MacTel), progression to severe non-proliferative (NPDR) and proliferative diabetic retinopathy (PDR) is rarely reported. We report multimodal imaging features of sight-threatening diabetic retinopathy (STDR) in eyes with MacTel.

**Methods:**

Retrospective case series of seven participants of the MacTel Study at the Moorfields Eye Hospital NHS Foundation Trust study site and one patient from the Institute of Retina and Vitreous of Londrina, Brazil. Sight threatening diabetic retinopathy was defined as severe NPDR, PDR or diabetic macular edema.

**Results:**

We report imaging features of 16 eyes of eight patients (7/8, 87.5% female) with diagnoses of MacTel and type 2 diabetes mellitus with STDR. Mean (SD) age was 56 (8.3) years. Patients were followed-up for a mean time of 9.1 (4.7) years. A total of 10/16 (62.5%) eyes showed PDR and 2/16 (12.5%) eyes presented a macular epiretinal neovascularization.

**Conclusions:**

People with diabetes mellitus and MacTel may not be protected from STDR as previously reported. Although the two diseases rarely co-exist, regular monitoring for diabetic retinopathy progression is recommended according to baseline retinopathy severity grades in line with established international guidelines. The presence of MacTel may not modify extended screening intervals, but there is no current evidence. The limited case series in the literature support treatment for complications and should follow the standard of care for either condition. Due to dual pathology, reactivation may be difficult to diagnose on standard imaging and multimodal imaging is recommended.

## Introduction

Macular telangiectasia type 2 (MacTel) is a bilateral retinal neurodegenerative disease with characteristic retinal vascular alterations. Clinical findings are restricted to an oval-shaped area centered around the fovea with a temporal epicenter, and include vascular telangiectasia, right-angled vessels, photoreceptor atrophy, and subretinal neovascularization. In patients with MacTel, a higher prevalence of Type 2 diabetes mellitus (DM2) and systemic hypertension as compared to the general population has been reported [1]. 

According to a recent systematic review and meta-analysis, around 26% of Europeans with diabetes type 1 and 2 have any level of diabetic retinopathy (DR), 2% have severe non-proliferative diabetic retinopathy (NPDR) and 2.2% require treatment for proliferative diabetic retinopathy (PDR) [2]. Globally, more than 1 in 10 people have diabetes mellitus and 30 to 40% have DR. Population surveys for MacTel have ranged from prevalence estimates of 0.06% in African populations, 5–23 per 100 000 people in Australia and 1 in 1000 in the Beaver Dam Eye study with a published incidence estimate of 0.0008% (95%CI, 0.4–1.2) per year in a white USA population [3]. Traditionally, panretinal photocoagulation (PRP) has been the standard of care for PDR with neovascularization of the disc (NVD) or elsewhere (NVE).

Previously, it has been suggested that features of advanced diabetic retinopathy occur at a lower prevalence in patients with MacTel as compared to patients with DM2 without MacTel. For instance, Jhingan and colleagues conducted a study analyzing a large MacTel cohort of 951 patients, and showed that out of 277 patients with diabetes (29%) only 28 eyes of 14 patients (5%) had DR, three eyes presented with PDR and only two eyes with initial mild NPDR progressed to PDR (1%) [4]. In a Dutch MacTel cohort entailing 206 eyes of 103 patients, Van Romunde and colleagues reported that of 49% of patients with diabetes only one patient (1%) had moderate NPDR in both eyes, while no patients met the criteria for severe NPDR or PDR [5]. The authors hypothesized that MacTel could be protective against the progression of DR.

Recently, a new fibrovascular epiretinal feature, distinctive from NV in PDR, so called epiretinal neovascularization (ERN), has been described in patients with MacTel [6]. Intraretinal pigment in MacTel has been shown to contain blood vessels on histopathology. The proximity of this vascular complex to the internal limiting membrane and vitreous may be a key pathogenic mechanism that differentiates the ERN phenotype from classic neovascularisation associated with diabetic retinopathy [7]. Little is known about the natural history of ERN, it remains unclear whether ERN will respond to anti-angiogenic or PRP treatment in the same way as NVE.

We sought to describe multimodal imaging characteristics in a case series of 16 eyes of eight participants with MacTel and STDR, including neovascular complications like PDR and ERN.

## Methods

MacTel was diagnosed based on multimodal imaging characteristics previously described [1], multimodal imaging included fluorescein angiography (FA, Optos, Optos plc, Dunfermline, Scotland, UK), color fundus photography (CFP, Topcon, Topcon Corporation, Tokyo, Japan), spectral domain optical coherence tomography (OCT, Heidelberg Engineering, Heidelberg, Germany), fundus autofluorescence (AF, Heidelberg Engineering, Heidelberg, Germany), confocal blue light reflectance (BLR, Heidelberg Engineering, Heidelberg, Germany), and optical coherence tomography angiography (OCTA, Zeiss Angioplex, Carl Zeiss Meditec, Inc., Dublin, USA). Diabetic retinopathy was graded according to the Royal College of Ophthalmology diabetic retinopathy guidelines [8]. Sight threatening diabetic retinopathy was defined as severe NPDR, PDR or diabetic macular edema (DME). Visual acuity (VA) is reported in Snellen acuity (feet). The patients presented in case 1 to 7 were identified and followed in medical retina clinics at Moorfields Eye Hospital and participated in the MacTel project clinical registry study. Briefly, the MacTel project (https://www.lmri.net/mactel/the-mactel-project/) is a research endeavor conducted by the MacTel Research Group and sponsored by the Lowy Medical Research Institute, to elucidate the pathogenesis, to develop outcome measures for clinical trials, and to identify and test treatments for MacTel (MacTel study). Case 8 was identified and reviewed at the Institute of Retina and Vitreous of Londrina, Brazil as part of their standard care.

## Results

Upon review of 222 people with MacTel at Moorfields Eye Hospital (MEH) NHS Foundation Trust, a total of 98/222 (44.1%) had comorbid DM2, and 24/98 patients (24.5%) had confirmed DR. From this group, we identified 7 with STDR (See Table 1).

**Table 1:** Patient characteristics, MacTel, and diabetic retinopathy features

Case	Sex	Age (years)	Duration of DM2 (years)	Diabetic retinopathy features	Follow-up (years)
1	Female	41	3	IRMA	9
2	Female	51	10	Right IRMA, Left NVE and PRP	9
3	Female	50	2	Right NVD and PRP, Left ERN and PRP	12
4	Female	65	8	NVE and PRP OU	14
5	Male	61	not known	NVE and PRP OU	6
6	Female	56	not known	Right mild NPDR, Left NVE and PRP	4
7	Female	59	not known	Right DME, bilateral PDR and PRP	3
8	Female	65	20	IRMA and PRP	16

Type 2 diabetes (DM2), Intraretinal microvascular abnormalities (IRMA), Neovascularisations elsewhere (NVE), Panretinal photocoagulation (PRP), Bilateral (OU), Epiretinal neovascularisation (ERN), Non-proliferative diabetic retinopathy (NPDR), Proliferative diabetic retinopathy (PDR), Diabetic macular edema (DME).

### Case 1

**Fig. 1:**
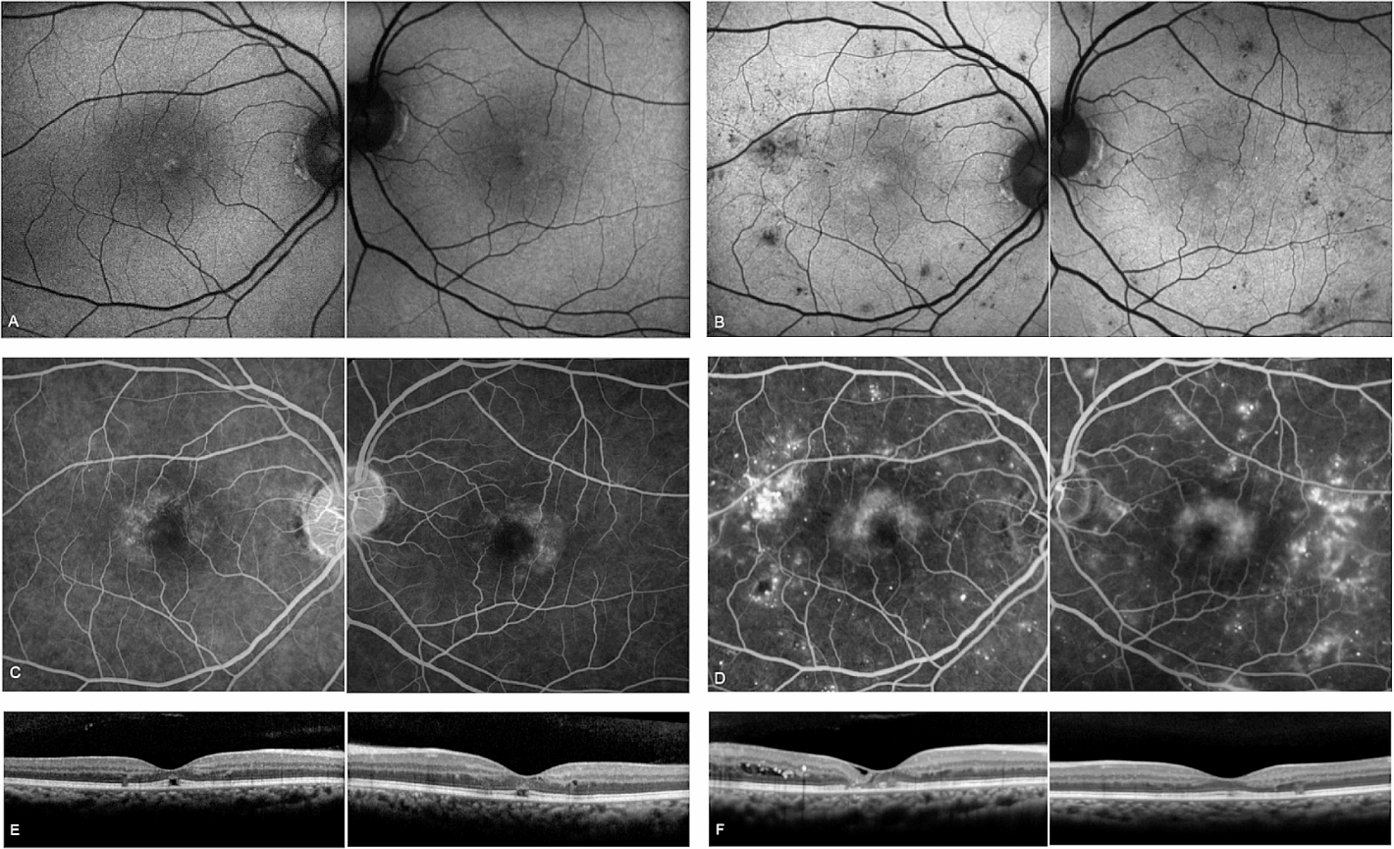
Blue light autofluorescence (AF), fluorescein angiography (FA) and optical coherence tomography (OCT). Comparison of AF (**A**, **B**) and FA (**C**, **D**) with progressing diabetic retinopathy (DR) in 2010 (**A**, **C**) and 2019 (**B**, **D**), and OCT in 2010 (**E**) and 2019 (**F**). Bilateral areas of non-perfusion, microaneurysms, and dot blot haemorrhages along with the MacTel signs: loss of macular pigment (centrally increased FAF) and perifoveal telangiectasia with late leakage on FA, (**C**) in early phase within first 90 s, (**D**) in late phase after 6 min. OCT at presentation (**E**) with subfoveal outer retinal ellipsoid zone dehiscence and early temporal outer retinal degeneration with left early cystoid spaces. At final visit (**F**) advanced subfoveal outer retinal atrophy, ILM drape and temporal intraretinal fluid at right side, with less affected outer retinal temporal changes in left eye

A 41- year-old female patient with a 3-year history of DM2 treated with oral medication presented in 2010 (9-year follow-up). Disease features typical for MacTel (right-angled vessels, temporal loss of retinal transparency (retinal greying) and hyperfluorescence on FA) and mild NPDR were noted in both eyes. Hemoglobin A1C (HbA1c) levels in the clinical records were unfortunately only noted as “high”. In 2019, DR had progressed, meeting severe NPDR criteria on FA, with intraretinal microvascular abnormalities (IRMA), areas of capillary non-perfusion, and multiple hemorrhages (severe NPDR). Hemorrhages and IRMA were very conspicuous on AF imaging (Fig. 1).

### Case 2

**Fig. 2:**
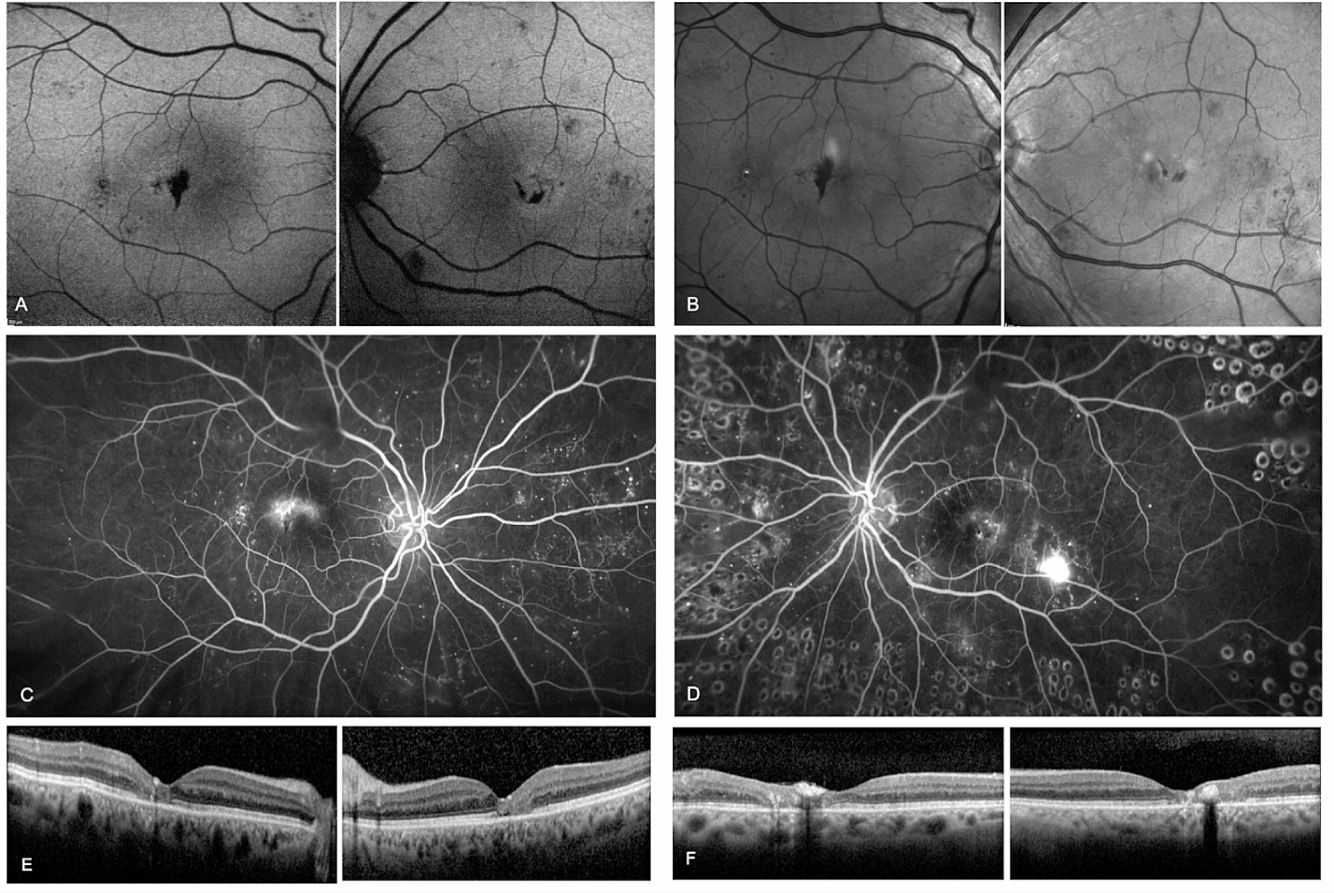
Blue light autofluorescence (AF) (**A**), confocal blue light reflectance (BLR) (**B**) and wide field fluorescence angiography (FA) (**C**, **D**) images taken at last follow up, with OCT at baseline (**E**) and last follow up (**F**). The AF (**A**) shows both increased and decreased central autofluorescence due to the loss of macular pigment in the parafoveal temporal area, intraretinal pigmentation and macular photoreceptor atrophy. Like case 1, signs of DR were very distinct as decreased AF. BLR (**B**) shows the disease specific oval shaped hyperreflectivity in the macular area, and also shows distinctly active NVE in the left eye. The wide field FA (**C**) shows parafoveal leakage typical for MacTel and leaking NV in the inferior temporal quadrant. Laser scars from previous PRP treatment are visible, FA taken after 2 min. OCT at baseline (**E**) with pronounced subfoveal and temporal outer retinal atrophy, and hyperreflective pigment clumping at last visit (**F**)

A 51-year-old female was followed between 2010 and 2019 (9 years). At first presentation with mild NPDR, she had a ten year history of DM2 (on oral medication and insulin) after her fourth pregnancy. Her glycaemic control deteriorated, from an HbA1c of 7.9% (62.8mmol/mol) in 2013, up to 12.9% (117.5 mmol/mol) in 2017. In 2016, ultra widefield FA showed NVE of the left eye and IRMA in the right eye. She underwent left PRP in multiple sessions. Repeated ultra widefield FA after nine months showed ongoing activity and fluorescein leakage in the left eye with subsequent further PRP, and severe NPDR in the right eye, VA was noted to be 20/40 in both eyes. Both AF and BLR also identified DR, including NVE (Fig. 2).

### Case 3

**Fig. 3:**
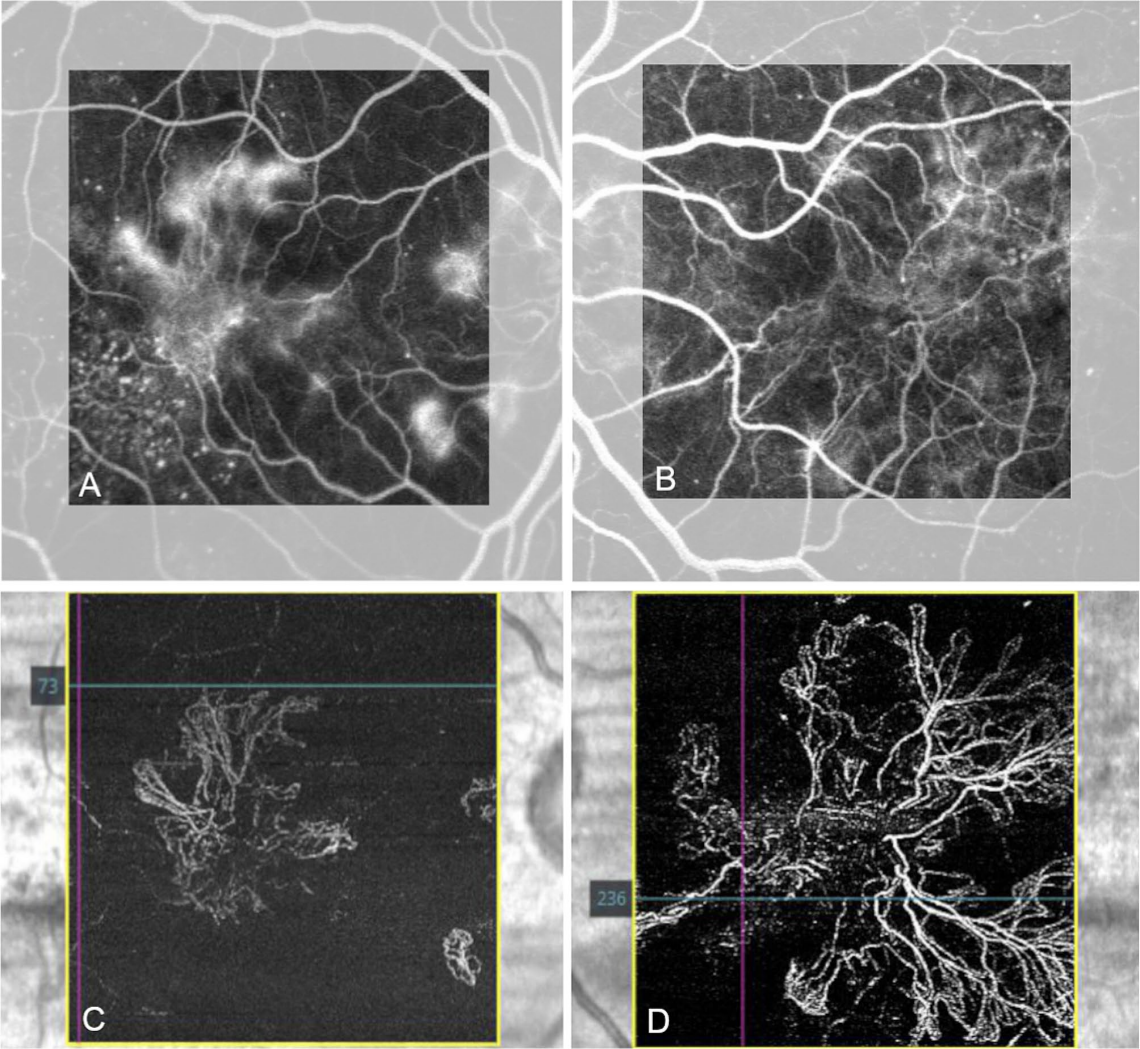
OCTA and fluorescein angiography (FA) from February, 2017. In both eyes FA shows diffuse leakage in the macular area after two minutes (**A**, **B**). On OCTA, extensive epiretinal neovascularization (ERN) becomes evident on the preretinal slab, left eye (**D**) more than right (**C**). Interestingly, the ERN is more visible on OCTA than on FA

**Fig. 4:**
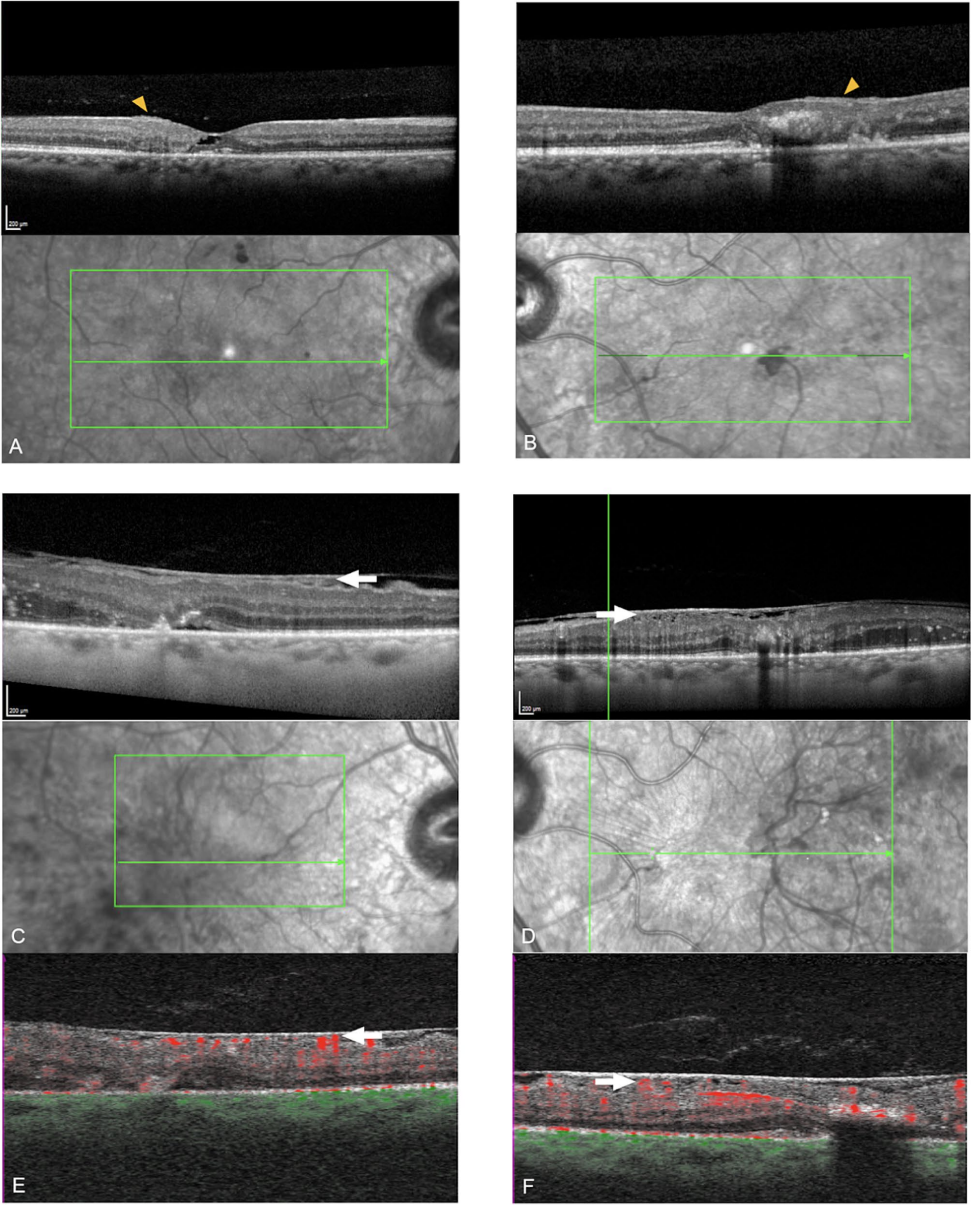
OCT, Near infrared and OCTA montage. Early signs of ERN development in 2014 in the right (**A**) and left (**B**) eye with subtle preretinal hyperreflective material adjacent to the internal limiting membrane (yellow arrowhead). In 2017 the patient developed right (**C**) and left (**D**) ERM after laser treatment with extensive ERN (white arrows). OCTA of the same location with flow signal underneath the epiretinal membrane (**E**, **F** -white arrows)

A 50-year-old female was followed between 2006 and 2018 (12 years). At first presentation with mild NPDR, she had a two year history of DM2 on oral medication. Visual acuity was reported as 20/20 in the right and 20/80 in the left eye. She underwent repeated FA and OCT. The HbA1c was recorded frequently, noting an increase up to 11.5% (102.2mmol/mol) in early 2014. At this time, the patient had suffered myocardial infarction, and fundus imaging only showed mild NPDR. The DR progressed in both eyes until late 2014, when she received PRP to treat neovascularization of the right disc (NVD) and the fovea, with additional focal laser for left diabetic non-center involving DME. Subsequently, the patient developed a bilateral epiretinal membrane. HbA1c in 2015 was noted to be 8.5% (69.4mmol/mol). In 2016, insulin was added to the diabetic management. In 2017, macular NV resembling ERN were diagnosed in the left eye based on FA and OCTA (Figs. 3 and 4), resulting in treatment with PRP and a course of five Aflibercept anti-VEGF intravitreal injections (IVI). Additional PRP was performed in the right eye, leading to bilateral regression in late 2017. In 2018, 12 years after the initial presentation, a bilateral reactivation of the macular NV defined as increase in size on OCTA compared to previous visits occurred which was treated with repeated anti-VEGF IVI, with a final VA of 20/80 Snellen in both eyes. OCT B-Scans demonstrate outer retinal disease and a marked ERM, related to the reduced VA levels (Fig. 4).

### Case 4

**Fig. 5:**
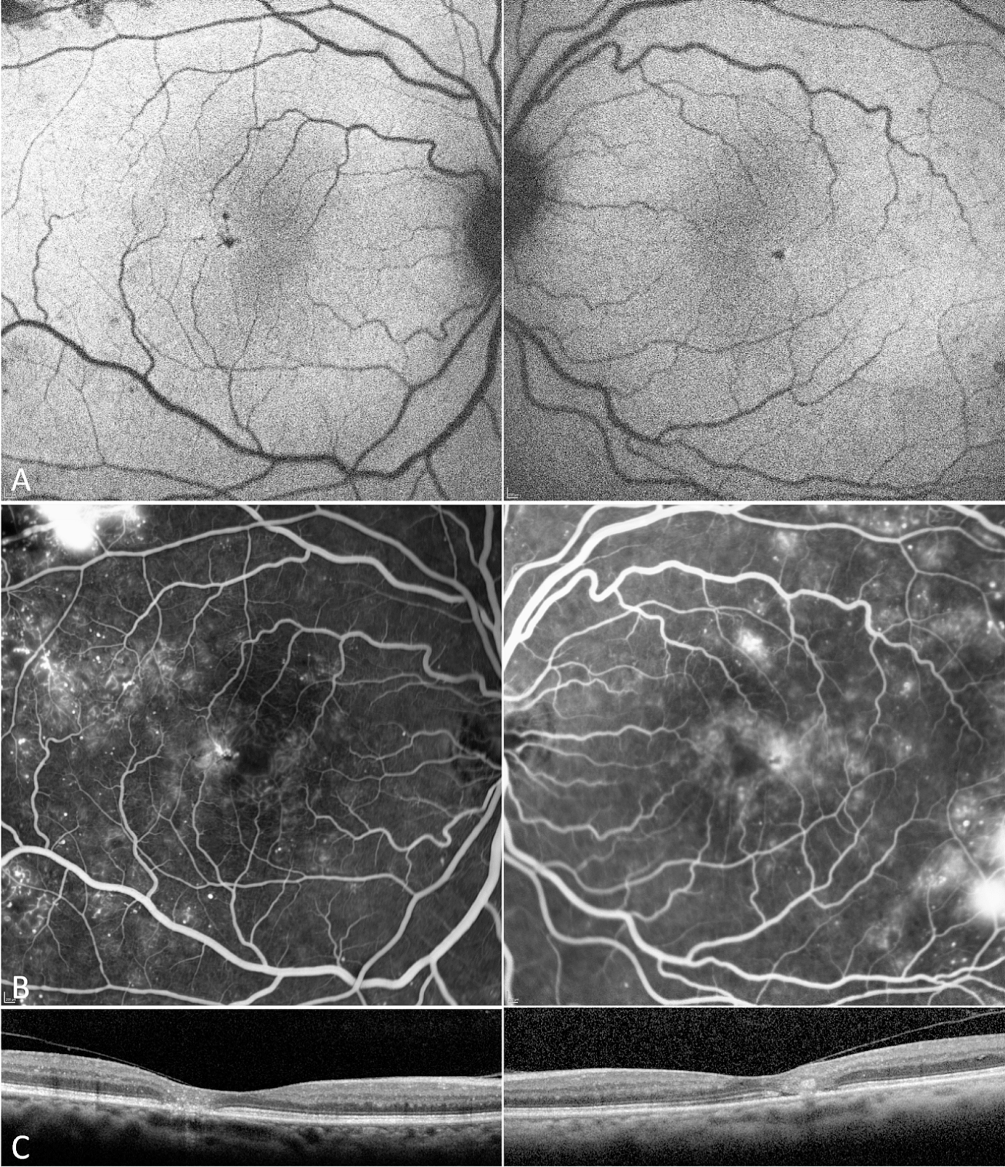
Blue light autofluorescence (AF), fluorescein angiography (FA) and optical coherence tomography (OCT) for right (right column) and left eyes (left column). AF (**A**) shows bilateral temporal wedge-shaped area of hyperautofluorescence and focal areas of lack of fluorescence temporal to the fovea typical of MacTel type 2, as supported by FA (**B**) findings showing temporal telangiectatic vessels in early phases with diffuse hyperfluorescence in the late phases. In addition, FA showed bilateral areas of non-perfusion, microaneurysms, and early leakage from an area in the superotemporal vascular arcade on the right eye, and an area inferotemporal to the vascular arcade on the left eye compatible with PDR. OCT at time of PDR detection (**C**) with outer retinal ellipsoid zone dehiscence and outer retinal degeneration temporal to the fovea in both eyes, left early cystoid space, and left temporal area of hyperreflectivity in the outer retina

A 65-year-old female patient with history of type 2 diabetes mellitus (8-year duration) treated with diet and oral medication, systemic arterial hypertension, and beta thalassemia trait was referred to MEH in 2008 from the English Diabetic Eye Screening Programme (DESP) for DME on the right eye. On initial examination, VA was 20/30 in both eyes and fundus biomicroscopy examination and macular OCT imaging revealed a presumptive diagnosis of bilateral mild NPDR with non-clinically significant DMO on the right eye. No macular changes were reported on the left eye. The patient attended annual follow-up visits and a diagnosis of bilateral MacTel in the context of mild NPDR was made in 2019, confirmed on secondary reviews of previous clinical and imaging findings (Fig. 5). During a MacTel study visit in 2022, VA was 20/50 bilaterally and fundus biomicroscopy and FA revealed the presence of NVE in both eyes, subsequently the patient underwent PRP in both eyes for PDR. There were no signs of anterior segment new vessels. (Fig. 5).

### Case 5

A 61-year-old male patient, initially referred for a presumed inherited retinal dystrophy, was reviewed between 2016 and 2022 (6 years). Cotton wool spots in the midperiphery with focal ischemia were noted on FA, with central macular atrophy and photoreceptor loss, hyperpigmentation and temporal thinning in both eyes on OCT scans. The right VA was noted as 20/250, the left as 20/40. In 2018, the patient was noted to have severe NPDR and high blood pressure (190/90mmHg) and further improvement of cardiovascular risk factors was initiated. The VA was 20/125 in the right and 20/80 in the left eye.

**Fig. 6:**
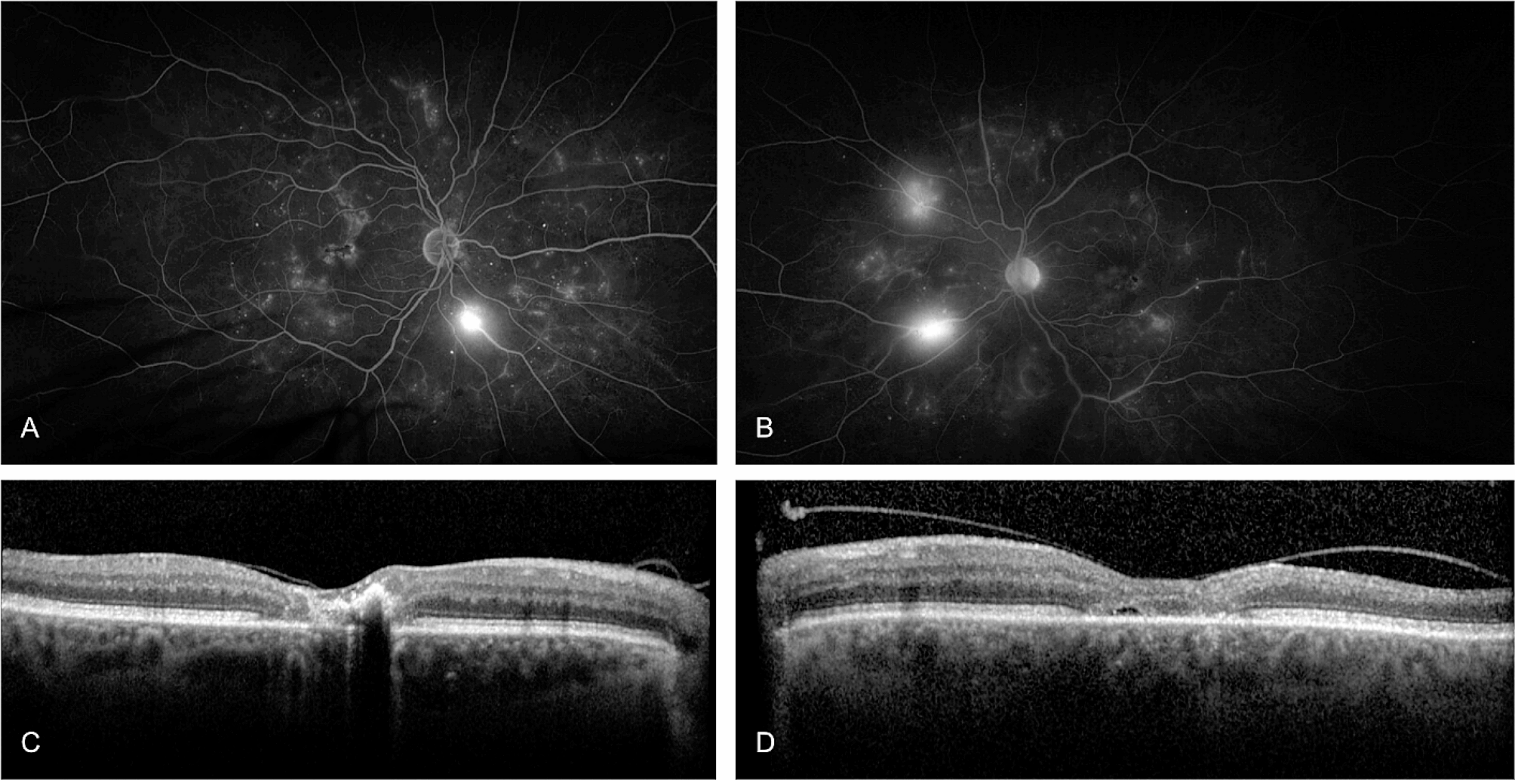
Fluorescence angiography FA (**A**, **B**), OCT (C,D). FA (**A**, **B**) from 2021, showing ultrawidefield imaging with inferonasal neovascularisations elsewhere (NVE), patchy areas of non-perfusion (ANP) in the midperiphery, with macular hypofluorescence in the macular area. OCT from 2021 (**C**) of the right eye shows hyperreflectivity due to pigment formation with temporal outer retinal loss and thinning,OCT of the left eye from 2021 (**D**) with subfoveal outer retinal loss, a small area of possible subretinal fluid and temporal thinning

In 2021, bilateral NVE were noted on a repeated FA, and the additional diagnosis of MacTel was made upon review of the old scans. Panretinal photocoagulation was initiated to both eyes. (Fig. 6)

### Case 6

**Fig. 7:**
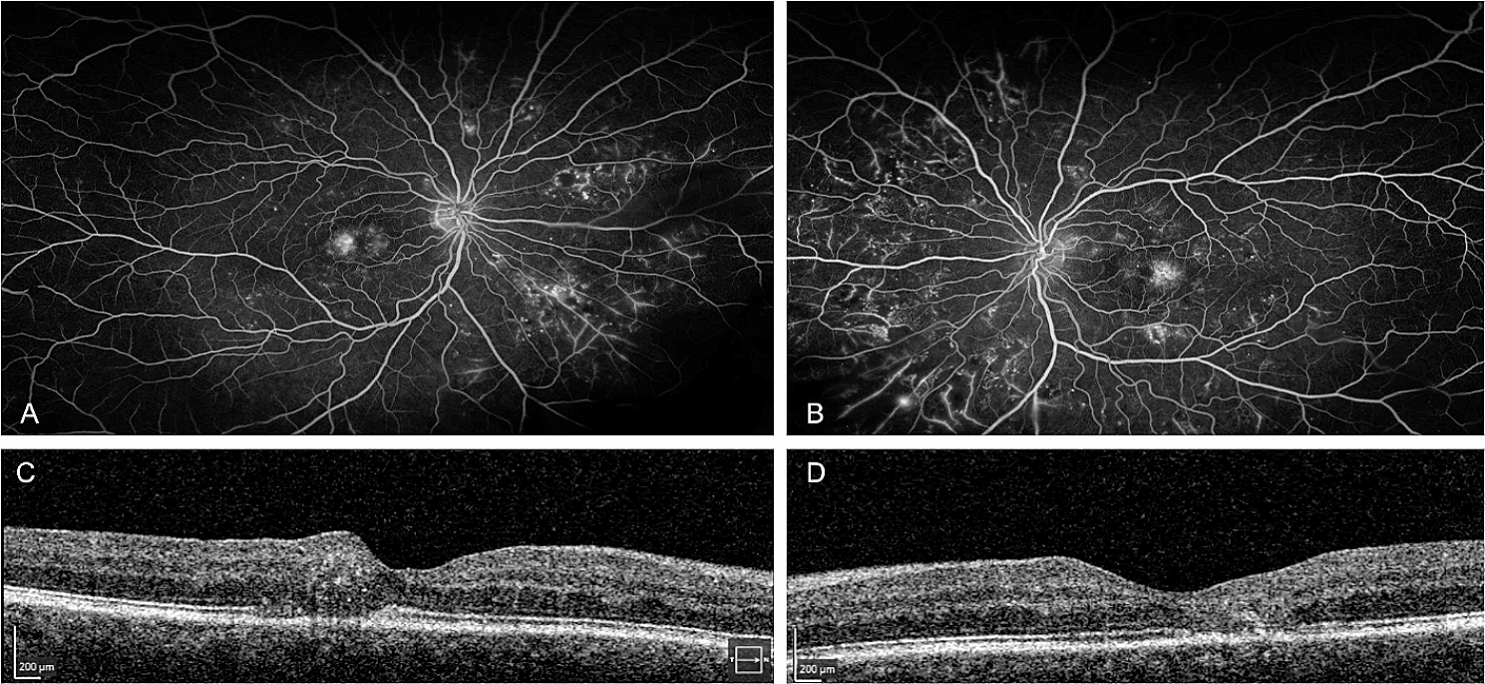
Ultra widefield fluorescein angiography (FA) and OCT. Ultra widefield FA of right (**A**) and left (B) eye from 2019 showing macular temporal hyperfluorescence due to telangiectatic changes, bilateral predominately nasal non perfusion (**A**, **B**) with IRMA in the right eye (**A**) and neovascularisations elsewhere inferonasally in the left eye (**B**). OCT from 2021 show typical MacTel features in both eyes (**C**, **D**) with temporal outer retinal loss with ellipsoid zone dehiscence, hyperreflective inner retinal changes and temporal thinning in the left eye (**D**)

A 56-year old female patient was diagnosed with right mild NPDR and left PDR in 2019, with a presenting VA of 20/40 to both eyes at University Hospitals Sussex NHS Foundation Trust. Fluorescein angiography revealed areas of non-perfusion, focal hyperfluorescence in the area of NVE and bilateral temporal diffuse macular leakage, consistent with Mactel. The patient was treated with left PRP. The HbA1c at the time was 6.9% (51.9 mmol/mol) and good diabetic control was emphasized. In 2020 the VA in the right eye deteriorated to 20/120, in 2021, further fill-in laser was recommended to the left eye due to NVE. The diagnosis of MacTel was established at a secondary appointment at MEH, where in addition to the right mild and left stable treated PDR and a right retinal greying, right angle vessel and temporal macular hyperpigmentation was noted, presenting as a hyperreflective mid retinal lesion with shadowing and a left outer retinal loss with temporal thinning and extrafoveal DME. (Fig. 7)

### Case 7

A 59-year old female patient was referred by the Northwest London DESP for bilateral PDR in 2020. Past ocular history entailed bilateral axial myopia, presbyopia, and mild nuclear cataracts. Past medical history was significant for essential hypertension, hypercholesterolemia, mixed anxiety and depressive disorder, myocardial infarction and smoking. Hemoglobin A1c levels were measured as 9.2% (77.05 mmol/mol) at presentation.

**Fig. 8:**
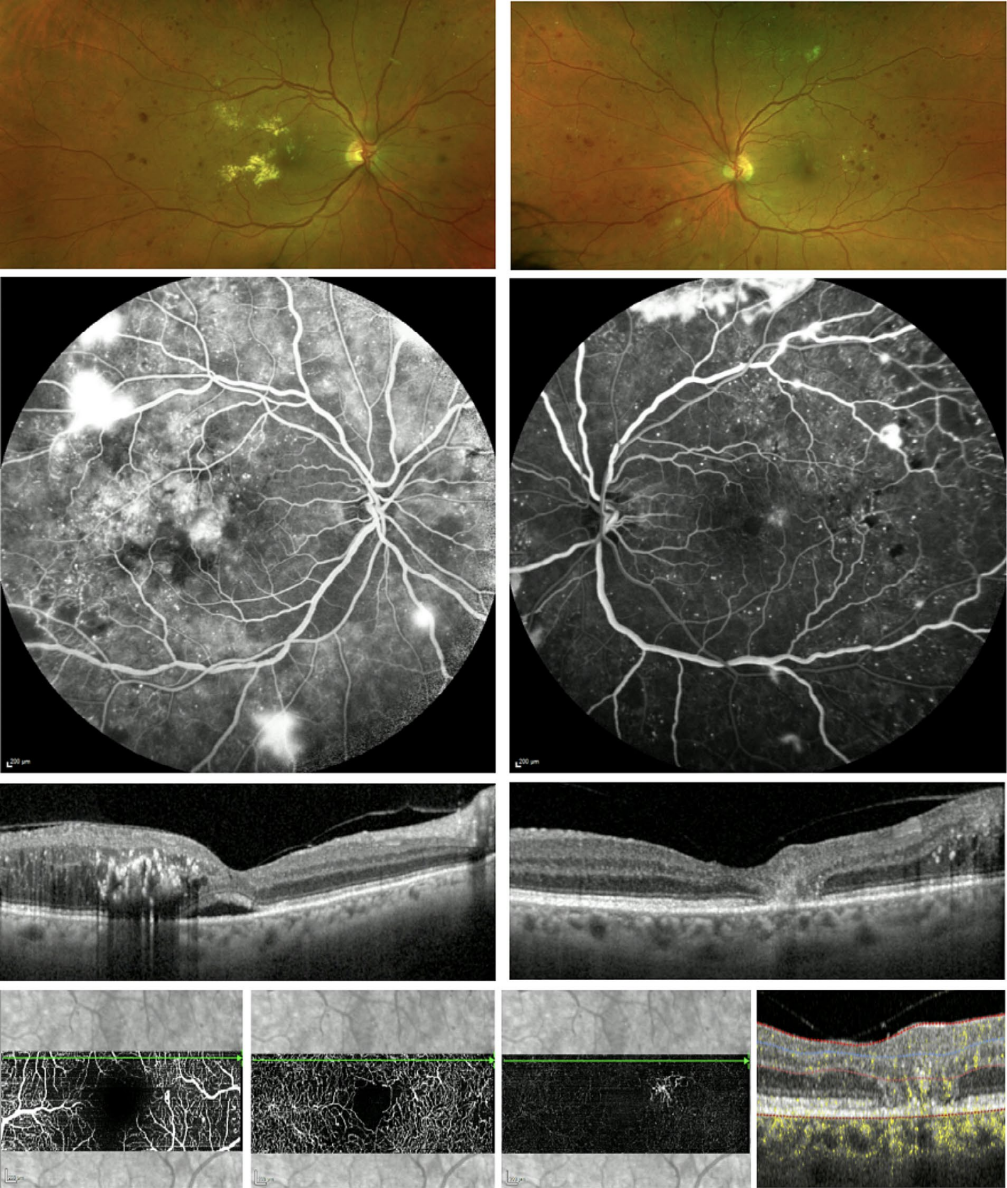
Optos widefield fundus imaging of the right (**A**) and left eye (**B**), showing features of proliferative diabetic retinopathy (PDR), such as new vessels along the temporal vascular arcades, venous beading and loops, dot and blot hemorrhages, and hard exudates. Fluorescein angiography at 12 min in the right (**C**) and at 1 min in the left eye (**D**) showing both features of PDR such as capillary non-perfusion, leakage from new vessels, and microaneurysms, as well as features from Macular Telangiectasia Type 2 (MacTel2), such as temporal para- and perifoveal leakage from telangiectatic, right-angled vessels. Optical coherence tomography (OCT) of the right eye (**E**) showing subretinal fluid, temporal macular thickening, intraretinal fluid and hyperreflective dots corresponding with hard exudates on fundus imaging. Optical coherence tomography (OCT) of the left eye (**F**) shows focal outer retinal atrophy and angular hyperreflectivity of the Henle fiber layer. En face optical coherence tomography angiography (OCTA) of the superficial (**G**), and (**H**) deep capillary plexus, and (**I**) the avascular segmentation slab showing telangiectatic right-angled vessels, anastomoses and rarefaction of the surrounding capillary bed in line with MacTel2

Visual acuity was measured as 20/60 in the right eye with no improvement with pinhole and 20/40, improving to 20/30 with pinhole in the left eye, multimodal imaging showed right intraretinal fluid with hyperreflective foci in the temporal macula with subfoveal fluid and left hyperautofluorescent macular changes with temporal macular pigment loss, OCT scans demonstrated right temporal intraretinal fluid with hyperreflective foci and subfoveal fluid, and left foveal thinning with EZ dehiscence and temporal parafoveal focal hyperreflectivity. Fluorescein angiography showed right superior macular leakage with inferior hypofluorescence, and left telangiectatic temporal macular microvascular alteration. Bilateral areas of non-perfusion perfusion and multiple areas of increasing hyperfluorescence in both eyes. (Fig. 8) A diagnosis of left MacTel, right DMO and bilateral PDR was established, intravitreal antiVEGF treatment to the right eye and bilateral PRP was initiated. Subsequently the patient underwent 5 IVI to the right eye and multiple PRP sessions to both retina. Visual acuity at the last visit was 20/40 in both eyes.

### Case 8

A 65 years-old female patient with a history of DM2 for 20 years presented to the Institute of Retina and Vitreous of Londrina, Brazil first in 2001 and was followed up for 16 years. The initial diabetes management consisted of oral medication, and later included insulin. HbA1c was reported as 8,2% (66.12 mmol/mol).

**Fig. 9:**
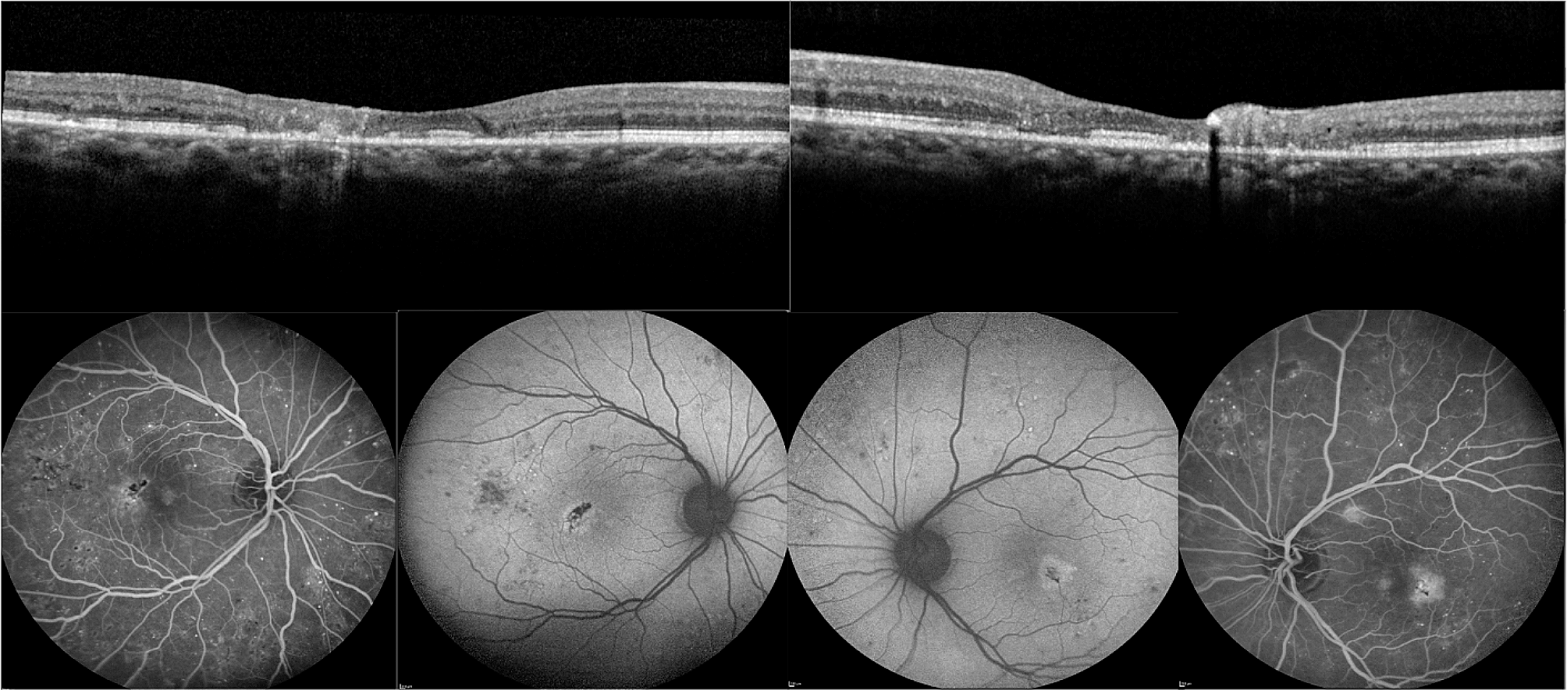
OCT scans of case 6 showing disorganization of internal retina as well loss of outer limiting membrane and photoreceptor layers (Fig. 6A and B).The fluorescein angiogram shows in both eyes retina non-perfusion ares, diffuse microaneurysms, leakage in the macular area and scatter photocoagulation spots (Fig. 6C and E).The autofluorescence shows proeminence of laser spots and the parafoveal hyperpigmentation (Fig. 6D and F). This examination was performed in 2012

The presenting VA was noted as 20/30 RE and 20/25 LE. The patient presented again in 2012 with severe NPDR and subsequently had bilateral PRP. The right VA was 20/40, in line with the patient’s complaints of deteriorating VA, and the left VA was recorded as 20/20. OCT scans confirmed bilateral outer retinal changes with pigment clumping, temporal thinning. Fluorescein angiography identified IRMA, peripheral non-perfusion and typical temporal macular hyperfluorescence as well as evidence of temporal macular grid laser. The diagnosis of MacTel in the presence of severe NPDR was established and a supplementary peripheral laser was performed (See Fig. 9).

## Discussion

### Main findings

In this case series, we report clinical and multimodal findings of sight threatening DR in eight patients with MacTel. Four patients participated in the MacTel study at MEH, where progression from mild to severe NPDR or PDR was observed. In four patients, the diagnosis of MacTel was made after STDR had already been treated. Furthermore, to our knowledge, bilateral ERN with concomitant signs of typical PDR elsewhere in the retina as presented in our case series has not yet been reported in MacTel.

### Findings in the context of the current literature

It has been described that DM2 is more common in patients with MacTel than in the general population [1]. Esposti and colleagues even suggested that the progression of DR in MacTel patients was slower and a protective or disease-modifying role of MacTel in DR has therefore been hypothesized [4, 5, 9]. The first four patients in our case series showed very mild DR at first presentation and throughout the first years of observation; systemic glucose control was poor in all three cases at the time of STDR and showed variable control during the period of follow up. Case 3 had suffered severe systemic cardiovascular complications but had stable mild DR around that time. This patient developed a rapid progression of DR with improvement of glycemic control. This phenomenon of worsening of DR after tightening of the systemic control is known as “early worsening” and has been described after the initiation of oral medication, insulin or bariatric surgery. Although its mechanisms are not yet fully understood, an upregulation of VEGF has been suggested [10]. The 24.5% point DR prevalence in patients with MacTel seen at MEH is higher than previously reported [4, 5]. Nevertheless, this is only a marginally lower prevalence of DR than reported in similar populations with diabetes [11]. 

We also present four cases where DR was deemed to explain both macular and peripheral retinal changes. The delayed diagnosis of MacTel may be explained by the coexistence of microcystic degeneration typical for MacTel in presence of the more prevalent findings of DR on OCT, late leak on FA and macular pigmentary changes interpreted as previous laser treatment. These cases may serve as examples of superimposed diseases complicating the diagnostic process.

It has been shown that duration of DM2 itself is a major risk factor for advanced DR, corresponding to a 42% risk accretion with each 5-year increase of disease duration after adjusting all other risk factors [11]. Furthermore, blood pressure and hyperglycemic control reflected by HbA1c are regarded as an important prognostic markers [10]. Both our cases with proliferative disease had rapid deterioration of glycemic control prior to the development of proliferative complications.

Only recently, ERN has been described as a rare feature in MacTel on OCTA [6]. Early signs include flat irregular hyperreflective material adjacent to the ILM in association with adjacent intraretinal pigment, which in due course mature into an ERN complex with flow signal on OCTA. In its original description, the authors hypothesized that the pigment location just underneath the ILM would cause ERN rather than choroidal neovascularization. Case 3 of this series is to our knowledge the first case with co-existing signs of PDR including NVD and simultaneous ERN. The extent of the neovascular complex may be attributed to the DR, as it is a microvascular disease and the drive for PDR may stem from VEGF secretion [12]. 

Intravitreal anti-VEGF injections have been used to treat secondary neovascularization in MacTel. Its use in non-proliferative MacTel has been investigated, showing only mild reduction of the intraretinal leakage with no change in VA [13]. The same group reported in a follow-up study that four out of nine eyes which received ranibizumab for non-proliferative MacTel developed secondary NV over an observational period of six years [14]. The authors discussed a possible rebound phenomenon with secondary VEGF upregulation. Case 3 of our series has shown marked bilateral reactivation of NV after initial response to VEGF followed by a prolonged inactive period of 12 months, which may be explained by a delayed response to VEGF upregulation after combined PRP and anti-VEGF intravitreal injections in this rare combination of a neurodegenerative disease with coexisting proliferative diabetic features.

## Limitations

The main limitation of this case series is the small number of cases. Nevertheless our case series shows evidence of STDR in a group of patients previously believed to be “protected” from DR/STDR. A possible reason for lower numbers of DR in the MacTel population than in the general population with diabetes might be explained by ascertainment bias. For example, people who are seeking help for subtle visual problems due to MacTel may have generally better health-seeking behaviors. Moreover, the MacTel study by protocol excludes patients with more than mild diabetic retinopathy from enrolment into the study, so there may be an ascertainment bias a priori at baseline. Physicians may also not diagnose MacTel in the presence of STDR as the classical signs of the disease may be masked.

## Conclusion

This small case series could suggest that a putative protective effect of MacTel in the development of PDR may be overestimated and that the incidence of neovascular complications in a larger case with both MacTel and diabetes may be similar to a matched population with diabetes. Therefore, people with MacTel should undergo regular DR screening in line with the level of their baseline retinopathy and not with a reduced screening interval due to the presence of MacTel. Treatments for DR will achieve disease stability in people with MacTel, but the neovascular phenotype may be modified by both MacTel and by the treatment. Reactivation may therefore be more difficult to diagnose on standard imaging and may require a serial multimodal imaging approach.

## Data Availability

All data generated or analyzed during this study are included in this article and its supplementary material files. Further enquiries can be directed to the corresponding author.
