# Correction: Sight threatening diabetic retinopathy in patients with macular telangiectasia type 2

**DOI:** 10.1186/s40942-024-00546-4

**Published:** 2024-03-25

**Authors:** Josef Huemer, Tjebo FC Heeren, Abraham Olvera-Barrios, Livia Faes, Antonio M. B. Casella, Edward Hughes, Adnan Tufail, Catherine Egan

**Affiliations:** 1 Moorfields Eye Hospital, NHS Foundation Trust, 62 City Rd.,, EC1V 2PD London, UK; 2 Department of Ophthalmology and Optometry, Kepler University Hospital, Linz, Austria; 3 University College London Institute of Ophthalmology, London, UK; 4 Department of Surgery, Health Sciences Center, Londrina State University, Paraná, Brazil; 5 University Hospitals Sussex NHS Foundation Trust, Brighton, UK

**Correction: Int J Retin Vitr 10, 28 (2024)**

10.1186/s40942-024-00545-5.

Following publication of the original article [1], the authors identified an error in the first paragraph of the abstract.

The published incorrect version is as follows:

**Purpose** Although diabetes is highly prevalent in patients with MacTel, progression to severe non-proliferative (NPDR) and proliferative diabetic retinopathy (PDR) is rarely reported. We report multimodal imaging features of sight-threatening diabetic retinopathy (STDR) in eyes with macular telangiectasia type 2 (MacTel).

The correct version should read:

**Purpose** Although diabetes is highly prevalent in patients with macular telangiectasia type 2 (MacTel), progression to severe non-proliferative (NPDR) and proliferative diabetic retinopathy (PDR) is rarely reported. We report multimodal imaging features of sight-threatening diabetic retinopathy (STDR) in eyes with MacTel.

Further to this, the last sentence of the “Introduction” was erroneously duplicated.

The original article [1] is corrected.
